# The impact of training chemotherapy safety standards with a smartphone application on the knowledge, attitude, and performance of nurses

**DOI:** 10.1186/s12912-023-01199-8

**Published:** 2023-02-16

**Authors:** Zohreh Hojati, Fateme Goudarzi, Shirin Hasanvand, Nasrin Galehdar, Mehdi Birjandi

**Affiliations:** 1 Social Determinants of Health Research Center, Boroujerd school of Nursing, Lorestan University of Medical Sciences, Khorramabad, Iran; 2 Social Determinants of Health Research Center, School of Nursing and Midwifery, Lorestan University of Medical Sciences, Khorramabad, Iran; 3 Surgical Technology Department, Paramedical Faculty, Lorestan University of Medical Sciences, Khorramabad, Iran; 4 Nutritional Health Research Center, Lorestan University of Medical Sciences, Khorramabad, Iran

**Keywords:** Chemotherapy, Education, Mobile application, Nurse, Safety

## Abstract

**Background:**

Nurses who play the leading role in caring for patients, especially nurses in the chemotherapy department who are constantly exposed to high-risk drugs and their side effects, should pay more attention to occupational safety. This study was performed to determine the effect of training chemotherapy safety standards using a smartphone application on nurses’ knowledge, attitude, and performance.

**Methods:**

The whole enumeration of fifty oncology nurses was recruited who they worked in 3 hospitals affiliated with Lorestan University of Medical Sciences (west of Iran). The study was conducted from June to November 2021. The training was done for four weeks with a smartphone application, including six main courses of familiarity with hazardous drugs, Personal Protective Equipment, preparation, storage and transfer, spilling, and wastes disposal of hazardous drudges . The nurses’ knowledge, attitude, and performance questionnaire were completed before, immediately after, and one month after the intervention. Data analysis was performed using SPSS version 26, descriptive and inferential statistical tests of independent t-test, one-way analysis of variance, Spearman’s rank correlation coefficient, repeated measures analysis of variance, and the Generalized Estimating Equation (GEE) model.

**Results:**

Mean knowledge score of participants before, immediately after, and one month after the intervention was (47.18 ± 8.19), (60.08 ± 3.82), and (61.88 ± 3.45), respectively. The mean attitude score of participants before, immediately after, and one month after the intervention was (30.34 ± 3.94), (34.32 ± 3.25), and (34.98 ± 2.88), in order, and the mean performance score of participants before, immediately after, and one month after the intervention was (43.60 ± 5.11), (51.78 ± 3.15) and (52.88 ± 3.06), respectively. The mean nurses’ knowledge, attitude, and performance score increased significantly over time (*P* < 0.001).

**Conclusions:**

Teaching chemotherapy safety standards using the application improved oncology nurses’ knowledge, attitude, and performance. Appropriate educational programs, especially by new methods such as E-learning, are recommended for providing safety for nurses.

## Background

Today, cancer is one of the leading health concerns of human societies [1]. The number of 19.3 million new cancer cases and approximately 10 million deaths were estimated worldwide in 2020 [2]. According to the estimation reported by the World Cancer Observatory in 2020, in Iran there has been 131,191 new cases of cancer in both sexes (70,704 Men and 60,487 Women) as well as 79,136 people died due to cancer [3]. Chemotherapy is the most common treatment for many cancers [4]. However, anticancer drugs are also teratogenic, mutagenic, and carcinogenic agents for humans [5]. Oncology nurses, who play the most critical role in the care of cancer patients, have the most exposure to antineoplastic drugs during the preparation, injection, transfer, storage, and elimination of waste [6, 7]. Exposure to these drugs may cause side effects such as primary DNA damage [8], increased risk of breast and rectal cancer [9], the presence of mutagenic agents in the urine [10], and adverse effects on the reproductive system [11], and skin disorders [12] for nurses. International organizations such as the National Institute for Occupational Safety and Health (NIOSH), the American Society of Hospital Pharmacists (ASHP), the Oncology Nursing Society (ONS), and the Occupational Safety and Health Administration (OSHA) recommend standard guidelines to prevent and minimize occupational accidents of health care providers [13, 14]. Despite the availability of these guidelines, many conducted studies report an inappropriate situation of observing safety standards by nurses in Egypt [14], Iran [15, 16], Iraq [17], and Cyprus [18], and show the need to implement the guidelines and training in this field in oncology units.

Non-compliance with safety proceedings when working with antineoplastic drugs can be due to nurses’ defects in knowledge, attitude, and performance (KAP). A study by Nwagbo et al. (2017) showed that oncology nurses needed to improve their knowledge in implementing standard guidelines [6]. People’s attitude can change their behavior and affect their safety culture directly or indirectly [19]. The third factor that indicates nurses’ non-compliance with safety standards is their performance with antineoplastic drugs. Gamal et al. [20] found that nurses had poor performance in this area and did not follow international standards properly. In Zayed et al. [14] study, two-thirds of nurses’ knowledge about the safe use of antineoplastic drugs was reported to be satisfactory, but only 36.4% of nurses had an appropriate performance. Koulounti et al. [18] also pointed out the need to implement educational programs with new techniques and methods to update nurses to protect them from antineoplastic drug side effects.

Various strategies have been used to improve the safety of nurses in oncology wards. In a systematic review performed by Crickman and Finnell [21], five methods were introduced to reduce antineoplastic drugs exposure, including the development of engineering controls, the use of Personal Protective Equipment (PPE), medical and environmental monitoring of common antineoplastic drugs, and risk assessment and training for health care providers using a comprehensive hazardous drugs (HDs) control program for improving the occupational safety of nurses.

Choosing the teaching method can be influenced by the existing facilities to achieve educational goals [22]. One of the most critical changes in education in the new age of information is the paradigm change from teacher-centered to inclusive education [23]. E-learning is overgrowing as a new method and an alternative to providing education in nursing [24]. Due to the interference of face-to-face retraining with nurse’s working hours or their leisure time, their lots of commitments, intensive shifts, and lack of motivation, using E-learning as an alternative or a complement method of traditional training can be a good approach for nurses’ retraining; Mobile learning is an emerging form of educational technology that complements learning outcomes by creating conversations, and sharing information and knowledge, and assisting colleagues and educators regardless of their geographical distance [25].

Surveys show that many studies have been conducted in the field of chemotherapy safety standards, including the study of Alehashem and Baniasadi [26] to evaluate the KAP of oncology nurses for the safe use of antineoplastic drugs in Iran, cross-sectional study byAsefa et al. [27] in Ethiopia, and the interventional study of Mishra et al. [28]. Most of the reviewed studies were non-interventional or interventional with educational methods such as lecture [22], video [29], and PowerPoint [28]. However, there was no study teaching these standards through E-learning. Thus, the present study aimed to detect the effect of teaching chemotherapy safety standards using a smartphone application on the KAP of oncology nurses.

## Method

### Design, setting, and sample

This semi-experimental one-group posttest-pretest study was performed on three hospitals affiliated with Lorestan University of Medical Sciences (LUMS)/ (west of Iran) from June to November in 2021. We used the total population sampling method. The population was the whole oncology nurses (57 nurses), from which 50 nurses were selected as a sample.

The inclusion criteria were as follows; to be Iranian nurses who speak in Persian language, employment in the chemotherapy wards, having at least a bachelor’s degree, not participating in a similar research project concurrent or before, and access to a smartphone. Also, in case of unwillingness to continue cooperating with the study, not attending the orientation meeting for getting familiar with the application, and changing the wards where nurses worked, the participants were excluded from the study. Two participants did not enter the study due to not having a smartphone. Five nurses were excluded because of being transfered from the oncology ward to other hospital unites.

### Data collection

We used a questionnaire developed by Alehashem and Baniasadi [26] to assess knowledge, attitude and perforamce (KAP) of oncology nurses in terms of safe handling of antineoplastic druges (ANPDs). This questionnaire is in Persian language and consistes of two below parts.

#### The part of demographic characteristices

This part of questionaire consistes of 13 questions about age, sex, marital status, level of education, employment status, nurse position, work experiences, work experiences in oncology unites, experiences of working with antineoplastic druges, membership in any oncology associations, the most common of work shifts (Morning, Evening or Night shifts), average working hours per week, and having previous refreshing courses in workplace.

#### Questionnaire to assess the KAP of nurses about oncology safety standards

This part of the questionnaire consistes of three components and 33 items totally. The commponets is in the 5-point Likert spectrum (strongly agree = 5, agree = 4, neutral = 3, disagree = 2 and strongly disagree = 1), which is completed as a self-report. The first component includes 13 questions to assess nurse’s knowledge about protocols and standards for the preparation, management, wasting disposal, and storage of antineoplastic drugs. The second section consists of 8 questions assessing nurse’s attitudes, feelings and concerns toward working at oncology unites. The last component contains 12 items evaluating participant’s performance in terms of preparation, management, wasting disposal, and storage of antineoplastic drugs. The mean scores were calculated for three sections of KAP, and the higher mean score indicated more agreement with the statements in the items. The mean score as cut-off point used to assess the level of KAP. This score was considered as 36 for knowledge with a range of 12–60, 24 for attitude with a range of 8–40, and 39 for performance with a range of 13–65. The scores greater than the mean score were considered sufficient for each components of KAP [26].

Content validity is a measure that shows how well an instrument measures all aspects of the desired construct. Two main types of Content Validity Index (CVI) are Item Content Validity Index (I-CVI) and Scale Content Validity Index (S-CVI). Moreover, a scale with excellent content validity should consist of I-CVIs of 0.78 or higher [30]. In the study by Alehashem and Baniasadi the content validity of the questionnaire was assessed by a panel of specialists, including two pharmacists, two toxicologists (with more than two years of experience in oncology units), and four oncology nurses (with more than five years of experience with antineoplastic drugs). The CVI-value was reported to be 0.78 which is acceptable. The internal consistency for different components of the instrument was measured separately using Cronbach’s alpha with a minimum internal stability requirement of 0.7 [26]. In this study, Cronbach’s alpha for knowledge, attitude, and performance were determined to be 0.87, 0.57, and 0.66, respectively.

### Intervention

#### Pre-intervention: design and development of a smartphone application

The design process of educational application was carried out from March to June 2021 in two steps: 1. Developing and evaluating the content, and 2. Building and evaluating the application. It is worthy to note that Iranian community as well as all Iranian nurses are single-nationality. Therefore, the application desgined in just Persian version.

##### 1. Developing and evaluating the content

One researcher (Z.H) extracted the scientific content of the software from reliable sources, including high-risk drug training protocols and standard instructions on how to work with these drugs. The scientific content was compiled based on reference books, recent articles, Occupational Safety and Health Administration (OSHA) standard guidelines, and nursing interventions of Iran’s National Standard Organization [13, 31–36]. The structure and the sequence of the content of the software was determined based on the following sections:

- Familiarity with hazardous drugs: types of cancer treatment, chemotherapy, ways of exposure to HDs, and complications of exposure with HDs
- Personal Protective Equipment: the necessity of using PPE, gown, head covering, foot covering, gloves, mask, protective glasses, face protection
- Handling of hazardous Drugs: physical conditions for drug preparation, drug preparation equipment
- Storage and transfer of hazardous drugs: transfer or storage of prepared drugs and half-used vials
- Spilling of hazardous drugs: leakage kit, necessary measures in case of leakage into the environment, necessary measures in case of cytotoxic contamination in biological cabinet or isolator
- Disposal of hazardous drudges wastes
- References

Many images and videos were used to make the educational content more exciting and compelling, adapted to the standard instructions. The content validity was confirmed by an oncologist, three nursing faculty members with the experiences of theoretical and clinical training in oncology, and an expert of educational design. Disagreements were discussed and exchanged until a consensus was reached. Following the necessary modification in the application’s content, the experts again approved the final version.

##### 2. Building and evaluating the application

In this step, the team firstly provided a paper-based design of the format of the application. Therefore, based on the educational purposes and content defined in previous step, the team determined the order and structure of the content as well as the location of them. The consequences of the design stage was the components considered in the homepage of the application detailed in below:

- The logo of Lorestan University of Medical Sciences: the research environments were oncology department affiliated with this university
- An access menu (including the items of *About us*, *App information*, *Content us* and *Exit*)
- Six icons for the main content included of *Hazardous Drugs* (*HDs*), *Personal Protective Equipment* (*PPE*), *Handling of hazardous drugs*, *Storage and transfer of hazardous drugs*, *Spilling of hazardous drugs* and *Disposal of hazardous drudges wastes*
- The icon of *References*

**Fig. 1:**
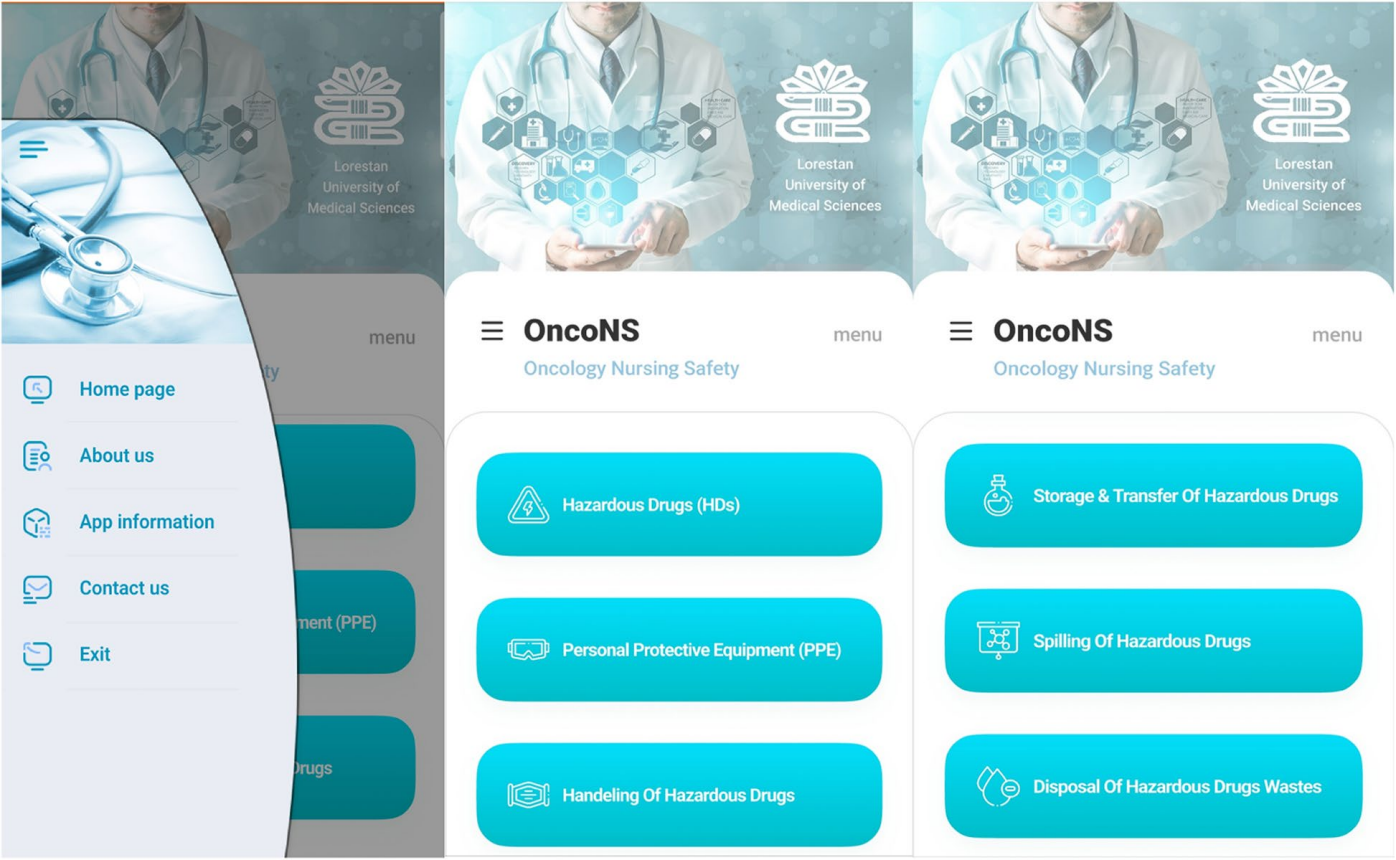
Photos of the OncoNS application

Then based on paper-design of the application, a web designer specialist provided the application programming with Android language in the Android Studio environment based on Java. Since the cellphones of nurses were based on either Android or IOS platform, the application were prepared for both mentioned platforms. OncoNS was chosen for the app’s name, which stands for Oncology Nursing Safety. User-friendliness principles were considered in developing the software. To do this, the application was designed for installation with just one click and in a short time. Moreover, the users could work with the application in a simple and straightforward way. Users could quickly move between pages and click on each part of the content without needing technical information. Camtasia Studio used to dub the videos from English to Persian is a strong and popular application for professional and fast modification of films, video recording and constructing the educational videoes. In the application, all videoes were addressed by putting the Uniform Resource Locator (URL) next them. It was done to observe the ethical consideration and facilitate to access the original version of videoes. Futhuremore, in designing the application we used the opinions of a panel of experts and their comments were discussed until a consensus was reached. Finally, the expert panel approved the final version of the application (Fig. 1).

### The intervention

**Fig. 2:**
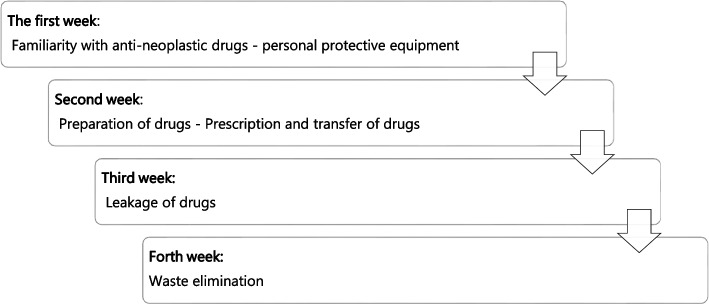
The schedule flowchart for studying and observing the application

A 20-min face-to-face meeting was held with each participant before the intervention. At this meeting, they completed a questionnaire that assessed nurses’ knowledge, attitude, and performance regarding safety standards in oncology units. Next, the application was installed on the participants’ mobile phones with the help of the researcher. Explanations about the environment of the application were presented to them. After receiving the application, participants had four weeks to read it and view its content (Fig. 2). Nurses received reminders by texting message or phone call every week between 17:00 p.m. and 19:00 p.m. Immediately after four-week intervention and one month after finishing the intervention, nurses were assessed for their knowledge, attitude, and performance related to oncology safety standards by completing the questionnaire in self-report way and in-person again. Indeed, the questionnaire was fullfiled in three points of time included before, immediately after the intervention, and one month after the intervention.

### Data analysis

After collecting and entering the data in SPSS software version 26, the ratios, central tendency, and dispersion index were reported. We used the independent t-test, analysis of variance, and Mann-Whitney U to compare the mean KAP score of nurses before the intervention in terms of occupational and demographic characteristics. The repeated measures test was used to compare the mean changes in KAP scores of nurses before, immediately after, and one month after the intervention. The Generalized Estimated Equations (GEE) were used to model the effect of the intervention on the KAP score by adjusting other variables. The KAP score using the Spearman correlation coefficient determined the correlation between occupational and demographic characteristics. A significance level of 5% was reported.

## Results

Overall, 50 of 55 eligible nurses worked in three oncology departements afilliated to LUMS participated in this study. All of them cooperated with doing reseach in all stages included intervention and fullfiling the questionnaire in mentioned three pointes of time. All participants competed the questionnaires completely. 92% of participants were female. The participants had a mean age of 29.88 ± 6.36 years and an average hospital work experience of 5.11 ± 5.00 years. Other demographic and educational characteristics of the participants are shown in Table 1.

**Table 1:** Demographic and occupational characteristics of the participants

Variable	N (%)^**a**^
	M ± SD^**b**^
**Sex**	
Women	46 (92)^a^
Men	4 (8)^a^
**Marital status**	
Single	28 (56)^a^
Married	22 (44)^a^
**Level of education**	
Bachelor Degree	49 (98)^a^
Master’s degree	1 (2)^a^
**Employment Status**	
Permanent employee	17 (34)^a^
Temporary employee	33 (66)^a^
**Nurse position**	
Nurse	47 (94)^a^
Chief	3 (6)^a^
**The Oncology Association membership**	
Yes	7 (14)^a^
No	43 (86)^a^
**The most common of work shifts**	
Morning	26 (52)^a^
Evening	9 (18)^a^
Morning- Evening	11 (22)^a^
Morning- Night	4 (8)^a^
**Having previous refreshing courses**	
Yes	44 (88)^a^
No	6 (12)^a^
**Age**	29.88 ± 6.36^b^
**Work experience (years)**	5.11 ± 5.00^b^
**work experiences in oncology unites (years)**	2.28 ± 1.74^b^
**Experiences of working with antineoplastic druges (years)**	2.28 ± 1.74^b^
**Average working hours per week**	47.08 ± 7.57^b^

N (%)^a^: Qualitative variables, M ± SD^b^: Quantitative variables

Based on univariate analysis, the mean attitude score of nurses with permanent employee official and contractual employment status in the baseline was significantly higher than that of temporary employee (*p* = 0.031). Also, in the baseline, there was no significant difference in the mean scores of nurses’ knowledge, attitude, and performance based on their sex, marital status, employment status, oncology association membership, the most common of work shifts, and Having previous refreshing courses (*p* > 0.05) (Table 2).

**Table 2:** Mean comparison of nurses’ knowledge, attitude, and performance based on demographic characteristics at the baseline

Variable	Knowledge	Attitude	Performance
	M ± SD	M ± SD	M ± SD
Sex			
Women	47.17 ± 8.15	30.32 ± 3.94	43.50 ± 5.21
Men	47.25 ± 9.94	30.50 ± 4.43	44.75 ± 4.34
*P*.Value	0.986	0.934	0.644
Marital status			
Single	45.46 ± 6.62	29.42 ± 3.39	42.60 ± 4.23
Married	49.36 ± 9.55	31.50 ± 4.35	44.86 ± 5.92
*P*.Value	0.095	0.064	0.123
Employment status			
Permanent employee	50.88 ± 10.99	32 ± 4.87	44.82 ± 5.82
Temporary employee	45.27 ± 5.59	29.48 ± 3.11	42.96 ± 4.68
*P*.Value	0.062	0.031	0.229
The Oncology association membership			
Yes	52.28 ± 11.20	32.42 ± 4.85	47.42 ± 6.42
No	46.34 ± 7.43	30.00 ± 3.72	42.97 ± 4.67
*P*.Value	0.218	0.132	0.120
The most common of work shifts			
Morning	47.50 ± 9.63	31.26 ± 4.45	44.15 ± 6.04
Evening	45.44 ± 6.40	28.77 ± 3.23	41.88 ± 4.16
Morning- Evening	47.90 ± 7.14	29.72 ± 3.34	42.54 ± 2.29
Morning- Night	47 ± 5.59	29.50 ± 2.38	46.75 ± 5.56
*P*.Value	0.917	0.355	0.357
Having previous refreshing courses			
Yes	47.11 ± 8.35	30.00 ± 4.06	43.70 ± 5.13
No	47.66 ± 7.60	32.83 ± 1.16	42.83 ± 5.38
*P*.Value	0.879	0.099	0.700

*M* Mean, *SD* Standard deviation

The Spearman correlation coefficient was used because the data distribution was not normal. There was a weak significant positive correlation between nurses’ age with knowledge score (*P* = 0.006) and attitude (*P* = 0.049) before the intervention, but the correlation between age and performance was insignificant. Also, a weak significant positive correlation was seen between nurses’ work experience with knowledge scores (*P* = 0.02). The correlation between nurses’ work experience and each of attitude and performance separately was insignificant (*P* > 0.05). There was a weak significant positive correlation between nurses’ work experience in the oncology ward with knowledge score (*P* = 0.04), but no statistically significant correlation was seen between attitude score and performance (*P* > 0.05). Also, there was a weak significant positive correlation between nurses’ work experience with antineoplastic drugs and knowledge score (*P* = 0.03). No significant correlation was seen between other variables with KAP scores before the intervention (*P* > 0.05) (Table 3).

**Table 3:** Correlation of baseline scores of knowledge, attitude, and performance with demographic characteristics of the participants

Base score / Variable	Knowledge		Attitude		Performance	
	***r***	***P***	***r***	***P***	***r***	***P***
Age (Year)	.38	.006*	.28	.049	**.07**	**.62**
Work experience (Year)	.32	.02*	.26	.07	**.03**	**.83**
Work experiences in oncology unites (years)	.29	.04*	.19	.179	**.23**	**.108**
Experiences of working with antineoplastic druges (years)	.30	.03*	.18	.199	**.23**	**.106**
Average working hours per week	_ .11	.456	.19	.189	**.10**	**.480**

*p < .05

There was a significant difference between the mean score changes in nurses’ knowledge over time (*P* < 0.001). A significant increase was seen in the mean knowledge score during the periods before the intervention, immediately after the intervention (*P* < 0.001), and one month after the intervention (*P* = 0.001). Also, there was a significant difference between the mean score changes in nurses’ attitudes over time (*P* < 0.001). A significant increase was seen in the mean attitude score during the periods before the intervention, immediately after the intervention (*P* < 0.001), and one month after the intervention (*P* = 0.003). There was a significant difference between the mean score changes in nurses’ performance over time (*P* < 0.001). So that a significant increase was seen in the mean performance score during the periods before the intervention, immediately after the intervention (*P* < 0.001), and one month after the intervention (*P* = 0.001) (Table 4).

**Table 4:** comparison of knowledge, attitude, and performance mean score changes of nurses at different times

Variable	Before the intervention	Immediately after	One month after	***F***	***P***.Value
	M ± SD	M ± SD	M ± SD		
Knowledge	47.18 ± 8.19	60.08 ± 3.82	61.88 ± 3.45	146.77	.001*
Attitude	30.34 ± 3.94	34.32 ± 3.25	34.98 ± 2.88	109.78	.001*
Performance	43.60 ± 5.11	51.78 ± 3.15	52.88 ± 3.06	132.68	.001*

*Abbreviations*: *M* Mean, *SD* Standard Deviation

*p < .05

Then, Variables with *P* < 0.2 were considered for inclusion in the Generalized Estimated Equations (GEE) model as independent variables to adjust their effects over time on nurses’ knowledge, attitude, and performance scores.

The GEE model and the linear link function showed that the mean knowledge score increased to 12.90 and 14.70 in the times of immediately after and one month after, as well as the attitude score increased to 3.98 and 4.64. The performance score increased to 8.18 and 9.28 in comparison with the score before the intervention by adjusting the effect of age, sex, marital status, previous training, work position, and work experience,. All changes over time were statistically significant (*P* < 0.001) (Table 5).

**Table 5:** Modeling changes in the average score of knowledge, attitude and performance of nurses over time by adjusting side variables using the GEE model

Variable	Knowledge β[%95 CI] *P*.Value	Attitude β[%95 CI] *P*.Value	Performance β[%95 CI] *P*.Value
Time			
Before the intervention	Reference	Reference	Reference
Immediately after the intervention	12.9[10.91, 14.88] (< 0.001)	3.98[3.19, 4.76] (< 0.001)	8.18[6.85, 9.51] (< 0.001)
One month after the intervention	14.7[12.47, 16.92] (< 0.001)	4.64[3.92, 5.36] (< 0.001)	9.28[7.77, 10.78] (< 0.001)
Marital status			
Single	Reference	Reference	Reference
Married	0.021[− 2.24, 2.29] (0.985)	− 0.07[−1.6, 1.4] (0.92)	−0.99[−2.51, 0.53] (0.201)
Employment status			
Permanent employee	Reference	Reference	Reference
Temporary employee	0.65[−2.59, 3.89] (0.351)	1[−1.14, 3.14] (0.361)	0.904[−1.01, 2.82] (0.355)
The Oncology association membership			
Yes	Reference	Reference	Reference
No	–	−0.6[− 2.5, 1.3] (0.535)	−2 [−4.42, 0.41] (0.104)
Age			
–	0.07[−0.29, 0.4] (0.644)	0.25[0.01, 0.5] (0.039)	–
Work experience			
–	0.11[− 0.21, 0.43] (0.493)	−0.01[− 0.33, 0.3] (0.914)	–
Work experiences in oncology unites			
–	1.17[0.53, 1.81] (< 0.001)	0.42[−0.002, 0.84] (0.049)	0.86[0.17, 1.21] (0.01)

## Discussion

This study aimed to determine the effect of teaching chemotherapy safety standards on oncology nurses’ KAP using a smartphone application.

### Knowledge

The results of the present study showed that oncology nurses’ knowledge about the safe use of antineoplastic druges was sufficient before the intervention. This result contradicted some studies. Only 4% of nurses had sufficient knowledge in a study by Bolbol et al. [37] in Egypt. However, in line with the present study, Zayed et al. [14] in Egypt showed that 67.3% of healthcare providers who administered antineoplastic drugs had adequate knowledge. Due to the implementation of the intervention, an increase in nurses’ awareness was observed from before the intervention till one month after. In line with this finding, a study by Crickman and Finnell [29] in Washington showed that a 30-min online training video for all nurses in the ward effectively increased their knowledge about improving treatment methods and reducing antineoplastic drug exposure. Also, in an interventional study, Bolbol et al. [37] found that after holding nine training sessions in the workplace, there was a significant increase in the knowledge level. Also, Elsayed Mahdy et al. [38] in Egypt found in a semi-experimental study that teaching standard instructions for working with antineoplastic drugs positively affected staff awareness about the standard principles of chemotherapy. In this study, the training was done theoretically and practically using a booklet guide and explaining its content during four face-to-face sessions [38]. Also, in consistent with the present study, a study by Ferguson et al. [39] in Australia showed that training through an online learning module delivered by mobile phone improved nurses’ knowledge of atrial fibrillation and anticoagulation. In the study of Nishimwe [40] in Rwanda, training based on mobile learning application improved the knowledge of nurses and midwives in the field of postpartum hemorrhage management and neonatal resuscitation.

### Attitudes

The results of nurses’ attitudes toward the safe use of antineoplastic drugs were similar to the participants’ awareness. Alehashem and Baniasadi [26] in Iran, also reported the desired level of nurse attitude. Khan et al. [41] in a study in Pakistan reported a moderate level of nurses’ attitudes toward preparing and managing chemotherapy drugs. We can mention variables such as physical environment, personal characteristics, cultural environment, and attitude measurement tools as the reasons for these differences. Nurses’ reactions and behaviors towards various care issues generally influenced by various factors such as beliefs, values, customs, and culture indicate the type of nurses’ attitudes [42]. The findings of this study showed that training through mobile applications improved nurses’ attitudes toward chemotherapy safety standards before the intervention until one month after that. Along with the present study, Taghizadeh Kermani et al. [43] in Iran showed that holding a training course in the form of 30 h of lectures and practical training on the main concepts of nursing in oncology can improve nurses’ attitudes. In a study by Keat et al. [44] in Malaysia, a significant change in nurses’ attitudes toward the safe use of antineoplastic drugs was reported after a 9-month pharmacy-based intervention including a series of technical, educational, and managerial support measures consisting of administering antineoplastic drugs in a closed system, training courses, training workshops, and instruction updates that are consistent with the present study.

### Performance

The results of the present study showed that oncology nurses’ performance regarding the safe use of antineoplastic druges was sufficient before the intervention. Consistent with this finding, in the study of Alehashem and Baniasadi [26] in Iran, the nurse’s performance score was reported to be satisfactory. However, according to the results obtained in the study of Zayed et al. [14] in Egypt and the study of Keat et al. [44] in Malaysia, nurses had poor performance in this field and did not correctly follow international standards. Possible reasons for this difference could be the different places of studies, the presence or absence of necessary equipment in the workplace, different tools used to measure staff performance, and previous training of the samples. An improvement in the performance level of nurses was observed before the intervention until one month after. Bolbol et al. [37] in Egypt also stated that participating in the training sessions can improve nurses’ performance in working with antineoplastic drugs. Also, Al-Attar and Al-Gannem [17] in Iraq stated that training positively affects nurses’ performance. The interventional study of Mishra et al. [28] in India also showed that the use of PowerPoint and discussion methods had improved the nurse’s performance regarding the safe use of antineoplastic drugs.

The nurses’ training has been done traditionally in most studies in this field. Continuing Medical Education (CME) is an essential principle in nursing professionalization, which can effectively improve nurses’ performance; E-learning can play an essential role in continuing medical education programs [45]. Due to the benefits of modern education, such as reduced training costs, accessibility in any time and place, reduced communication costs, and so on [46, 47], the use of E-learning, especially mobile applications for nurses who are busy with work, is more convenient and accessible. In confirmation of the contents mentioned in the study of Khoshnoodifar et al. [46] in Iran, it was found that the E-learning method can increase nurses’ knowledge scores more than traditional education. Also, in the study of Farshbaf-Khalili et al. [48] in Iran, multimedia electronic training during 1-h sessions for 4 weeks was more effective than lecture training in increasing the knowledge, attitude and performance of nursing staff regarding pain management. Also, many studies have shown high satisfaction among people and positive education results in using E-learning alone or with traditional education in medical sciences [24, 45].

## Study limitations

The small sample size of the study due to the limited number of nurses in the chemotherapy unites was one of the study limitations. Furthermore, most of nurses had gotten training related to the chemotherapy hazardes and safety guidelines which is indispensable and is an crucial prerequisite to work with the chemical druge. Scince all nurses spoke in Persian languge, the application was designed just in Persian.

## Conclusion

According to the results of the present study, the training of chemotherapy safety standards using the OncoNS smartphone application had a desirable effect on the KAP of oncology nurses. Due to the effectiveness of the application and, on the other hand, the problems of nurses such as high workload, interference of face-to-face retraining with their working hours, leisure, and family issues, the use of E-learning method is recommended for training nurses and other health care providers. We hope that nursing managers help nurses improve their knowledge, attitude, and performance in occupational safety by using proper educational planning, especially new educational methods such as E-learning in the form of a smartphone application.

## Data Availability

Data is available upon reasonable request from the corresponding author.

## Abbreviations

KAP: Knowledge, Attitude, and Performance
HDs: Hazardous drugs
NIOSH: National Institute for Occupational Safety and Health
ASHP: American Society of Hospital Pharmacists
ONS: Oncology Nursing Society
OSHA: Occupational Safety and Health Administration
PPE: Personal Protective Equipment
CVI: Content Validity Index
I-CVI: Item Content Validity Index
S-CVI: Scale Content Validity Index
URL: Uniform Resource Locator
